# Establishment of a Tissue-Mimicking Surrogate for Pulmonary Lesions to Improve the Development of RFA Instruments and Algorithms

**DOI:** 10.3390/biomedicines10051100

**Published:** 2022-05-10

**Authors:** Louisa Bühler, Markus D. Enderle, Nicolas Kahn, Markus Polke, Marc A. Schneider, Claus Peter Heußel, Felix J. F. Herth, Walter Linzenbold

**Affiliations:** 1 Erbe Elektromedizin GmbH, 72072 Tübingen, Germany; louisa.buehler@erbe-med.com (L.B.); markus.enderle@erbe-med.com (M.D.E.); 2 Department of Pneumology and Respiratory Care Medicine, Thoraxklinik at Heidelberg University Hospital, 69126 Heidelberg, Germany; nicolas.kahn@med.uni-heidelberg.de (N.K.); markus.polke@med.uni-heidelberg.de (M.P.); felix.herth@med.uni-heidelberg.de (F.J.F.H.); 3 Translational Lung Research Center Heidelberg (TLRC), Member of the German Center for Lung Research (DZL), University of Heidelberg, 69120 Heidelberg, Germany; marc.schneider@med.uni-heidelberg.de (M.A.S.); hsl22@uni-heidelberg.de (C.P.H.); 4 Translational Research Unit, Thoraxklinik at Heidelberg University Hospital, 69126 Heidelberg, Germany; 5 Department of Diagnostic and Interventional Radiology, Heidelberg University Hospital, 69120 Heidelberg, Germany; 6 Department of Diagnostic and Interventional Radiology with Nuclear Medicine, Thoraxklinik at Heidelberg University Hospital, 69126 Heidelberg, Germany

**Keywords:** tissue-mimicking phantom, thermochromic, agar, radiofrequency ablation, electrical impedance, lung cancer, pulmonary lesions

## Abstract

(1) Development of radiofrequency ablation (RFA) systems for pulmonary lesions is restricted by availability of human tumor specimens and limited comparability of animal tissue. We aimed to develop a new surrogate tissue overcoming these drawbacks. (2) Reference values for electrical impedance in lung tumor tissue were collected during routine lung tumor RFA (*n* = 10). Subsequently, a tissue-mimicking surrogate with comparable electrical impedance and facilitating detection of the ablation margins was developed. (3) The mean electrical impedance for all patients was 103.5 ± 14.7 Ω. In the optimized surrogate tissue model consisting of 68% agar solution, 23% egg yolk, 9% thermochromic ink, and variable amounts of sodium chloride, the mean electrical impedance was adjustable from 74.3 ± 0.4 Ω to 183.2 ± 5.6 Ω and was a function (y = 368.4x + 175.2; R^2^ = 0.96; *p* < 0.001) of sodium chloride concentration (between 0 and 0.3%). The surrogate tissue achieved sufficient dimensional stability, and sample cuts revealed clear margins of color change for temperatures higher 60 °C. (4) The tissue-mimicking surrogate can be adapted to lung tumor with respect to its electrical properties. As the surrogate tissue allows for simple and cost-effective manufacturing, it is suitable for extensive laboratory testing of RFA systems for pulmonary ablation.

## 1. Introduction

With an incidence of 2.3 million new cases in 2020 and a mortality up to 80%, lung cancer is one of the leading cancer-related causes of death worldwide [1]. For resectable lung tumors, medical guidelines recommend surgical excision of pulmonary segments or entire lobes as first-line therapy [2,3]. However, in over 50% of patients, surgical excision is not feasible due to advanced cancer stages or comorbidities of patients, e.g., reduced expiratory volume or oxygen uptake [4]. Especially for those patients ineligible for surgical tissue resection, thermal ablation is a promising alternative due to its minimal invasiveness and preservation of surrounding healthy tissue. Radiofrequency ablation (RFA) represents one of the most common types of thermal ablation and has already been established in clinical routine, e.g., for ablation of hepatic tumors [5]. In pulmonology, RFA is currently recommended for early stage non-small cell lung cancer (NSCLC) without lymph node involvement and for distant metastases as a third-line treatment [3,6,7,8].

During a RFA procedure, the instrument is inserted percutaneously or endoscopically into the lung nodule, and subsequently, an electrical current is activated in order to induce tissue necrosis. To date, one of the main shortcomings is tissue charring at the electrode surface and thus limited ablation zone size [9], making RFA only applicable for smaller lung nodules. Larger ablation zones have been achieved by, e.g., internal electrode cooling and modulation of current flow algorithms [10,11,12,13,14].

Improvement and validation of advanced RFA instruments and its current flow algorithms require extensive laboratory testing; however, human lung tumor specimens are not available in large quantities and require specific handling, preparation, and storage. Furthermore, different in vivo animal lung tumor models have been used for testing and validation of RFA systems (e.g., mice, hamster, sheep) [15]. Especially ovine pulmonary adenocarcinoma (OPA) in sheep represents a suitable model due to high anatomical and physiological similarity of human and sheep lung [15,16]. However, tumor generation and subsequent ablation in vivo does not match the 3R principles [17] and is not cost- and time-efficient for the purpose of extensive testing and validation. Ex vivo animal tissue is more easily available, and especially bovine liver is a commonly used standard tissue for RFA settings [18,19,20,21,22] but transfer of ablation outcome to human lung tumor is restricted for example by different electrical conductivity and thus impedance.

Moreover, different tissue-mimicking surrogates, such as gel phantoms, have been used since they allow for the addition of further components that influence tissue properties [23,24,25,26]. The most common gelling agents such as gelatin, agar, and polyacrylamide, however, have different limitations such as low melting points, high opacity, low mechanical strength, or toxic ingredients [27]. In addition, they lack protein denaturation and thus tissue desiccation, and plain gel phantoms do not facilitate visualization of the area where 60 °C has been reached, indicating instant and irreversible tissue damage [28,29,30].

Therefore, in this study, we aimed to develop a new tissue mimicking human lung tumor surrogate for RFA, which has comparable electrical impedance to tumor tissue and facilitates detection of a 60 °C threshold.

## 2. Materials and Methods

### 2.1. Study Design

In this study, a two-stage approach was chosen: First, reference values for electrical impedance in lung tumor tissue were collected in vivo, and subsequently, a tissue-mimicking surrogate was developed. In the in vivo part of the study, which had been approved by the IRB (AZ 080/2006), 10 patients were enrolled for pulmonary RFA at the Thoraxklinik Heidelberg from 2017 to 2018. Included were patients with non-small cell lung cancer (NSCLC), specifically adenocarcinoma (7 patients) and squamous cell carcinoma (3 patients), ineligible for surgical resection and tolerable of anesthesia. In the second part of the study, a tissue-mimicking phantom was developed on the basis of the collected electrical impedance data in vivo. Different ingredients were analyzed for their properties during RFA, and the ablation behavior of the final surrogate composition was validated against bovine liver tissue, which is one of the commonly used tissues for evaluating RFA outcomes ex vivo [18,19,20,21,22].

### 2.2. Percutaneous RFA In Vivo

A bipolar, internally cooled RFA probe (Erbe Elektromedizin GmbH, Tübingen, Germany) was utilized for pulmonary RFA. Prior to ablation, the probe was inserted percutaneously into the lung of lung tumor patients (Thoraxklinik Heidelberg) and positioned centrally within the tumor mass under CT guidance. When optimal probe position had been assured, tumor ablation was performed using an electrosurgical unit with a working frequency of 350 kHz (VIO^®^300D, Erbe Elektromedizin GmbH), the corresponding current algorithm (BiTherm Mode), and a cooling unit (ERBECRYO^®^ 2, Erbe Elektromedizin GmbH). Power output settings were chosen according to the discretion of the surgeon, and electrical data including impedance was recorded using in-house-built software during the entire duration of ablation.

### 2.3. Establishment of an Adequate Surrogate

On the basis of the collected electrical data from the clinical setting, the requirements for a tissue-mimicking surrogate were defined. The major requirements included the possibility to obtain impedance curves similar to lung tumor tissue and to achieve an adjustable mean impedance level in the range of in vivo impedance, overall reproducibility, and visible color change of target tissue indicating a temperature threshold of 60 °C. Further characteristics considered favorable were moldability, easy manufacturing, and cost-efficiency.

In order to meet these requirements, a surrogate tissue consisting of sodium chloride (Merck, Darmstadt, Germany, LOT K0001004817), liquid thermochromic ink with a color change to magenta at 60 °C (LCR Hallcrest, Glenview, IL, USA, LOT: 1211KRO006004EKG), pure egg yolk, agar powder (Cleaver Scientific, Rugby, UK, LOT: 14180928), and demineralized water was tested. Initially, two different masses were fabricated: egg yolk was mixed with sodium chloride and thermochromic ink and separately, a 4% agar solution was manufactured using agar powder and demineralized water. The agar solution was heated stepwise to 100 °C and subsequently cooled down to 43 °C before both masses were mixed and stirred thoroughly. Afterwards, the mixture was poured into molds and cooled for several hours.

### 2.4. Impedance Measurement in Surrogate

Cubic tissue blocks with an approximate volume of 20 cm^3^ were cut from each mold. The RFA probe was inserted centrally within the tissue block, and ablation at room temperature was performed identically to percutaneous RFA in vivo, while impedance values were recorded during the entire ablation time using an in-house-built software program. Thereafter, the samples were cut along the instrument channel, and the long axis (along the instrument channel) and the short axis (perpendicular to instrument channel) of the ablated tissue were measured.

### 2.5. Comparison of Surrogate and Bovine Liver

**Figure 1:**
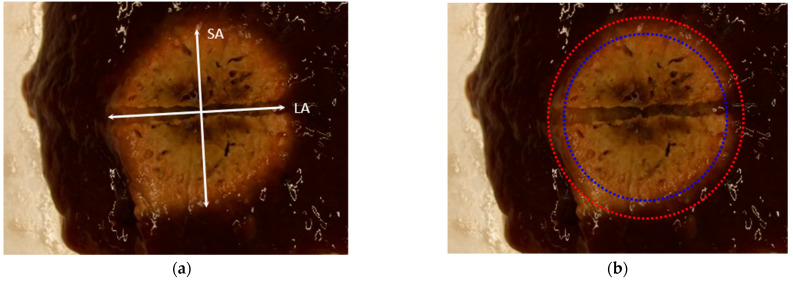
Assessment of lesion zone size within liver tissue samples post-ablation. (**a**) Determination of short axis (SA) and long axis (LA) and (**b**) and differentiation of the inner (blue) and outer (red) ablation zone.

To validate that the developed surrogate tissue can mimic ablation behavior of fresh tissue, ablation outcome was compared to the current lab standard of bovine liver. Fresh liver from a local butcher (Faerber, Balingen Germany) and the surrogate tissue were ablated for 150 s, 300 s, 450 s, and 600 s each. After ablation, the samples were cut along the instrument channel, and lesion size was measured along the short axis and long axis by one experienced evaluator. For liver tissue, the inner (darker) and an outer (brighter) zone of affected tissue were assessed separately since they represent different extents of cellular damage (Figure 1) [31].

### 2.6. Statistical Analysis

All data were evaluated and visualized using GraphPad Prism version 7 for Windows (GraphPad Software, La Jolla, California, USA, www.graphpad.com, (accessed on 1 January 2022)). Descriptive statistics were carried out to describe the basic features of the collected data. Differences between independent quantitative data was detected using a two-tailed Student’s t-test, provided that the data were normally distributed. The normal distribution was confirmed with a KS-test (Kolmogorov–Smirnov test). For analysis of non-normally distributed samples, the Mann–Whitney test was used. Simple linear regression was used to show the linear relationship between impedance and sodium concentration. *p*-values <0.05 were considered statistically significant.

## 3. Results

### 3.1. Impedance in NSCLC In Vivo

**Figure 2:**
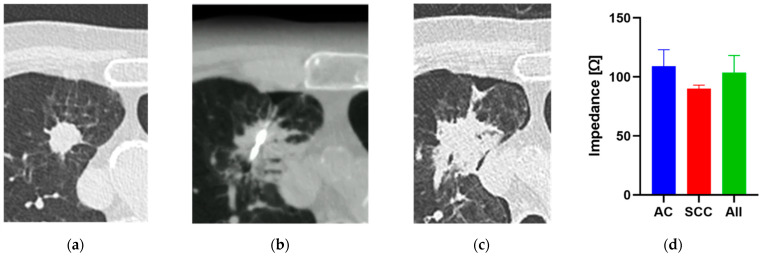
Example of a radio-frequency ablation (RFA) performed percutaneously using a bipolar probe. Displayed are (**a**) CT images before insertion of the RFA probe into the lung tumor mass, (**b**) the formation of necrotic tissue during RFA, (**c**) the formation of necrotic tissue after RFA, and (**d**) the recorded electrical impedance of the tumor (type: adenocarcinoma (AC) and squamous cell carcinoma (SCC)) throughout the procedure.

As a reference for surrogate development, the impedance during RF ablation (Figure 2) in NSCLC patients was investigated. The calculated mean impedance value for all patients (*n* = 10) was 103.5 ± 14.7 Ω. Although the mean value for adenocarcinoma patients (*n* = 7) was 109.2 ± 13.9 Ω, we observed a lower, but not statistically significant (*p* = 0.07), difference of impedance for squamous cell carcinoma patients (*n* = 3; 90.1 ± 3.0 Ω).

### 3.2. Evaluation of the Established Surrogate

**Figure 3:**
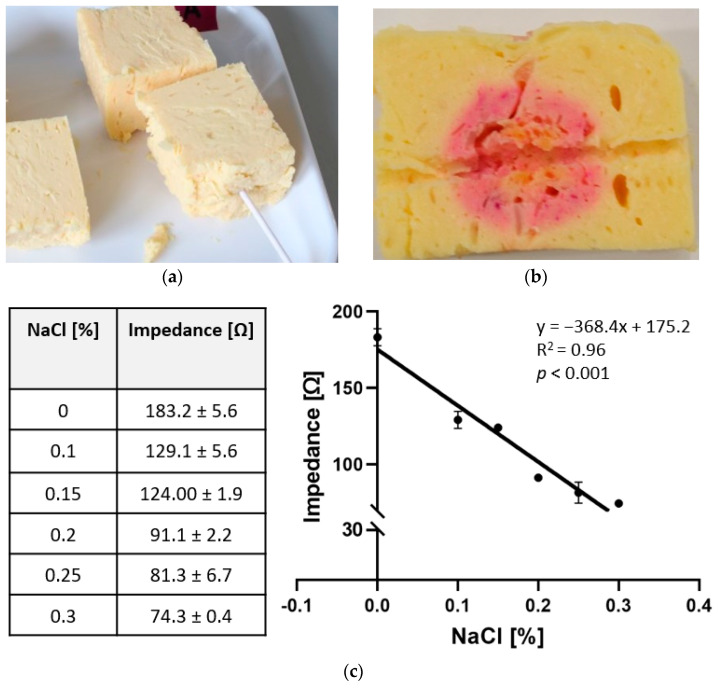
Evaluation of established surrogate. (**a**) Experimental setup with inserted RFA instrument into one piece of surrogate; (**b**) ablation outcome with color change from thermochromic ink after the block was cut along the probe channel; and (**c**) calculated mean impedance values for each sodium chloride concentration and linear fit of impedance as a function of sodium chloride.

Due to limitations of available tumor tissue, an adequate synthetic surrogate for human lung tumor tissue is of high interest. The herein developed tissue model consisted of 68% agar solution, 23% egg yolk, 9% thermochromic ink, and variable amounts of sodium chloride based on our own in vivo impedance measurements (Figure 3). Independent of the sodium chloride concentration used, the developed surrogate tissue showed sufficient dimensional stability and only slight melting in the area directly at the electrode surface. Sample cuts revealed clear margins of color change (Figure 3). Moreover, the impedance curves during ablation were comparable to in vivo ablations, showing stable plateaus and subsequent roll-offs after several minutes (data not shown). The average impedance of the surrogate tissue ranged from 74.3 ± 0.4 Ω to 183.2 ± 5.6 Ω and was a function of sodium chloride concentration (y = 368.4x + 175.2; R^2^ = 0.96; *p* < 0.001) for the herein presented surrogate composition (Figure 3).

### 3.3. Comparison of Surrogate and Bovine Liver Tissue

For the purpose of validation, ablation parameters in the surrogate were compared to the lab standard of bovine liver tissue in a total of 12 samples. For the surrogate tissue, 0.096% sodium chloride was used in order to match the electrical impedance of bovine liver tissue (140 Ω).

**Figure 4:**
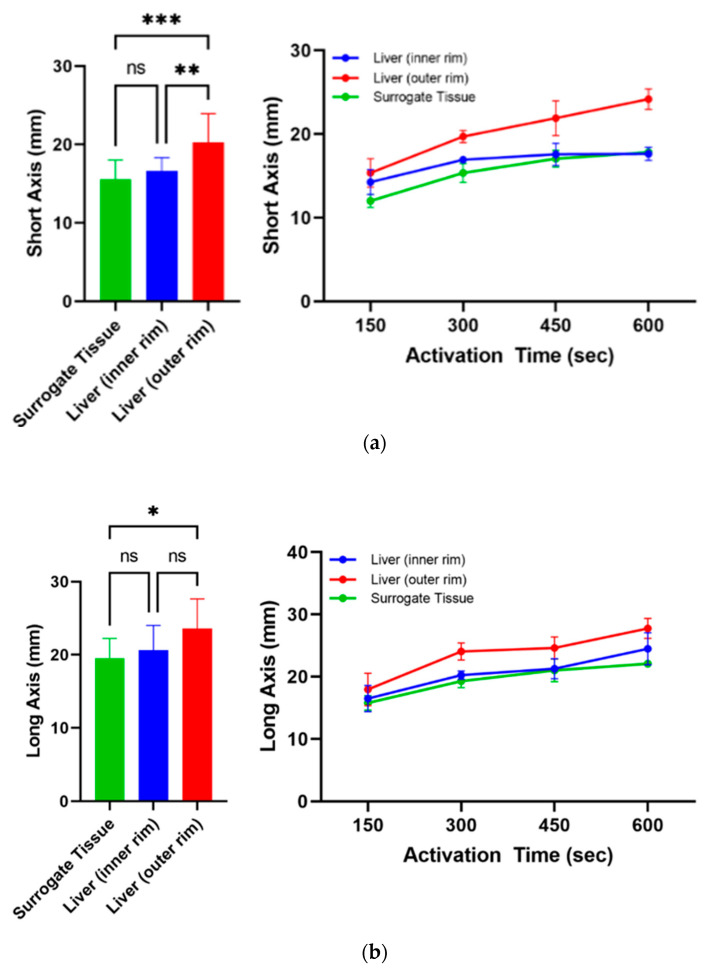
Comparison of ablation growth during RFA in surrogate tissue with 0.096% sodium chloride (green) to the inner (blue) and outer (red) rim of bovine liver as a mean over all ablation points (left) and as a function of RF activation time (right); (**a**) short axis and (**b**) long axis. * *p* ≤ 0.05; ** *p* ≤ 0.01; *** *p* ≤ 0.001; ns: *p* > 0.05.

In general, the surrogate tissue showed very similar growth behavior as bovine liver tissue for an activation time between 150 and 600 s (Figure 4). Especially the difference between the inner zone (mean SA: 16.6 ± 1.7 mm; mean LA: 20.6 ± 3.4 mm) of the ablation area in bovine liver and the surrogate (SA: 15.6 ± 2.5; LA: 19.5 ± 2.7 mm) was not statistically significant (SA: *p* = 0.617; LA: *p* < 0.720) with deviations in the range of only 0.2–2.3 mm in the short axis and 0.3–2.4 mm in the long axis. Compared to the outer rim of liver tissue (SA: 20.3.6 ± 3.6 mm; LA: 23.6 ± 4.0 mm), we observed a significant difference in both the short (*p* < 0.001) and long axes (*p* = 0.018) in the surrogate tissue.

## 4. Discussion

In this study, we developed a new tissue-mimicking surrogate for RFA that facilitates the detection of a 60 °C threshold and can be adjusted to different electrical impedances simulating different tissue types concerning their electrical properties.

Electrical tissue conductivity, which negatively correlates to tissue impedance, largely influences RF ablation. As described in the bioheat equation by Pennes [32] and Goldberg [33], the coagulation necrosis equals the energy deposited multiplied by local tissue interactions minus heat loss. Consequently, high electrical impedance and thus low amount of energy deposited results in limited tissue necrosis and vice versa. This, in contrast to microwave ablation, where ablation is induced by oscillation of water molecules in an electromagnetic field, is why electrical impedance plays a pivotal role in the assessment of RFA performance, and results from tissue-mimicking phantoms are only transferable in the case of similar electrical impedance.

Aqueous salt solutions, such as sodium chloride solutions, contain free ions that are able to conduct an electrical current. In the herein-developed surrogate tissue, a linear correlation of sodium chloride on electrical conductivity was observed. Since adjustment of the electrical impedance in the range of 75–180 Ω was feasible with the developed surrogate tissue, it can be adapted to the mean electrical impedance of lung tumor tissue determined in the in vivo part of the study (103.46 ± 14.68 Ω) or potentially different lung tumor types. Even if the difference of electrical impedance between adenocarcinoma and squamous carcinoma was statistically not significant in this study, a larger sample size might provide more precise electrical characterization and thus the possibility of corresponding adjustment of the surrogate tissue in the future.

The surrogate consists of commercially available components and facilitates easy and cost-effective manufacturing. We did not evaluate the amount and existence of gas bubbles in this study, but there was the possibility that small gas bubbles were existent in the surrogate matrix because of extensive stirring. However, as we did not observe any bubbles in the surrogate tissue during the whole study at the ablation site, we do not think that the bubbles altered the uniformity of the ablation zone to a large extent. Therefore, in contrast to human lung tumor tissue, which requires specific precaution measures and is not available in large quantities, the surrogate tissue is more suitable for extensive laboratory testing. Furthermore, the egg yolk and air bubbles included in the surrogate tissue induced desiccation and thus RFA roll-offs. Alternatively, the addition of egg white or bovine albumin serum (BSA) to gel phantoms has been reported in other studies; however, the coagulation temperature of such phantoms is reported above 70 °C, which is higher than the usual temperature threshold for irreversible protein denaturation of biological tissue [34,35,36].

A major advantage of the developed surrogate over natural tissue is the precise visualization of the achieved tissue temperature. Even if cells can be damaged at temperatures lower than 60 °C, only those guarantee instant and irreversible cell death [28,29,30]. The addition of thermochromic ink to the surrogate tissue enables distinct visualization of the tissue area where 60 °C has been reached during ablation, whereas natural tissue, e.g., bovine liver, typically shows an inner (darker) area and an outer (brighter) area. In this study, the size of the ablation zone in surrogate tissue was comparable to the inner area of ablated bovine liver. As it has been shown in CT/MRI and histological images, the outer rim of an ablated liver lesion only represents a zone of transient periablational hyperemia with edema and cellular damage, meaning that those cells are not necessarily devitalized and are able to regenerate after a few weeks [31,37]. Consequently, we suggest that assessment of the actual area of necrosis is reliable using the developed surrogate tissue by measuring the inner rim that the here-introduced surrogate model can provide.

A minor shortcoming of the developed surrogate tissue is the limited thermal stability of agar at temperatures higher than 85 °C. RFA can reach temperatures up to 100 °C, and consequently local melting at the electrode surface could influence the ablation behavior, although the temperature at the electrodes surface, which is in direct contact to the tissue, should not exceed 80 °C to prevent desiccation [38,39]. Some previous studies [34,35,40] describe the utilization of polyacrylamide gel, which has a significantly higher melting point; however, toxic ingredients and complex manufacturing limit its application for extensive laboratory testing.

## 5. Conclusions

The tissue-mimicking surrogate for RF ablation developed in this study can be adapted to lung tumor with respect to its electrical properties and comparable ablation outcome to the inner coagulation zone in bovine liver, reflecting irreversible tissue damage. Further, the introduced thermochromic ink enables the visualization of coagulation necrosis with a 60 °C margin. As the surrogate tissue allows for simple and cost-effective manufacturing, it is suitable for extensive laboratory testing of RFA instruments and algorithms for the purpose of pulmonary ablation. Moreover, the surrogate tissue does not include human or animal tissue and matches the 3R principles. We suggest that the developed surrogate tissue can not only be used as a substitute for lung tumor tissue but could also be adjusted to tumors of other organs in the future.

## Data Availability

Not applicable.
